# Altered Degranulation and pH of Neutrophil Phagosomes Impacts Antimicrobial Efficiency in Cystic Fibrosis

**DOI:** 10.3389/fimmu.2020.600033

**Published:** 2020-12-18

**Authors:** Elaine Hayes, Mark P. Murphy, Kerstin Pohl, Niall Browne, Karen McQuillan, Le Er Saw, Clare Foley, Fatma Gargoum, Oliver J. McElvaney, Padraig Hawkins, Cedric Gunaratnam, Noel G. McElvaney, Emer P. Reeves

**Affiliations:** Irish Centre for Genetic Lung Disease, Department of Medicine, Royal College of Surgeons in Ireland, Education and Research Centre, Beaumont Hospital, Dublin, Ireland

**Keywords:** ion channel potentiator therapy, pH, degranulation, bacterial phagosome, cystic fibrosis, neutrophils

## Abstract

Studies have endeavored to understand the cause for impaired antimicrobial killing by neutrophils of people with cystic fibrosis (PWCF). The aim of this study was to focus on the bacterial phagosome. Possible alterations in degranulation of cytoplasmic granules and changes in pH were assessed. Circulating neutrophils were purified from PWCF (n = 28), PWCF receiving ivacaftor therapy (n = 10), and healthy controls (n = 28). Degranulation was assessed by Western blot analysis and flow cytometry. The pH of phagosomes was determined by use of BCECF-AM-labelled *Staphylococcus aureus* or SNARF labelled *Candida albicans*. The antibacterial effect of all treatments tested was determined by colony forming units enumeration. Bacterial killing by CF and healthy control neutrophils were found to differ (p = 0.0006). By use of flow cytometry and subcellular fractionation the kinetics of intraphagosomal degranulation were found to be significantly altered in CF phagosomes, as demonstrated by increased primary granule CD63 (p = 0.0001) and myeloperoxidase (MPO) content (p = 0.03). In contrast, decreased secondary and tertiary granule CD66b (p = 0.002) and decreased hCAP-18 and MMP-9 (p = 0.02), were observed. After 8 min phagocytosis the pH in phagosomes of neutrophils of PWCF was significantly elevated (p = 0.0001), and the percentage of viable bacteria was significantly increased compared to HC (p = 0.002). Results demonstrate that the recorded alterations in phagosomal pH generate suboptimal conditions for MPO related peroxidase, and α-defensin and azurocidine enzymatic killing of *Staphylococcus aureus* and *Pseudomonas aeruginosa*. The pattern of dysregulated MPO degranulation (p = 0.02) and prolonged phagosomal alkalinization in CF neutrophils were normalized *in vivo* following treatment with the ion channel potentiator ivacaftor (p = 0.04). Our results confirm that alterations of circulating neutrophils from PWCF are corrected by CFTR modulator therapy, and raise a question related to possible delayed proton channel activity in CF.

## Introduction

Cystic fibrosis (CF) is an autosomal recessive disease, caused by mutations in the gene encoding the cystic fibrosis transmembrane conductance regulator (CFTR) anion channel (1), resulting in altered chloride ion (Cl^-^) transport. A lack of CFTR function affects multiple systems throughout the body, yet is characterized by structural lung disease with bronchiectasis from a very young age (2, 3), coupled with a severe dysregulated inflammatory response (4). Inflammation is further amplified by microbial infections of the airways, initially *Staphylococcus aureus (S. aureus)* in infants, and later *Pseudomonas aeruginosa (P. aeruginosa)* (5, 6). Neutrophils are one of the first immune cells to be recruited to the site of bacterial infection, and deﬁciency in function renders patients susceptible to chronic recurrent infections (7, 8). Studies have demonstrated that neutrophils account for ~70% of the total cell count in CF bronchial lavage fluid (9, 10) and free neutrophil elastase (NE) activity is detectable in airway samples of children with CF as young as 3 months old (2). Thus, in CF there are apparent contradictory conditions, whereby recruited neutrophils and infecting microbes co-exist in the airways. Our interest in CF was further fueled by the availability of specific therapeutics in CF such as the CFTR potentiator ivacaftor (VX770). This therapy is available for PWCF with the *Gly551As*p mutation and demonstrated a marked improvement in patient lung function (11) and decreased sweat Cl^-^ concentration to the normal range (12).

Within the blood circulation, CFTR is detectable on platelets (13), monocytes (14), and lymphocytes (15), and is present and functional on neutrophil membranes (16, 17). Consequently, neutrophil dysfunction in patients with CF has been investigated either due to inflammation or a lack of CFTR function. Studies have revealed alterations in degranulation (18–20), chemotaxis (21), recruitment (22), oxidant formation (23, 24) and apoptosis (25, 26). However, research demonstrating that only 25% of neutrophils generate neutrophil extracellular traps (NETs) against *S. aureus*, with the majority of bacterial killing occurring post phagocytosis (27), is suggestive of suboptimal CF phagosomal performance.

Upon engulfment of a bacterium, oxygen consumption increases by up to one hundred-fold, and transfer of electrons across the membrane of the phagosome by an NADPH oxidase, NOX2, results in intra-phagosomal superoxide $\left(O_{2}^{-}\right)$ production. This supports oxidative mechanisms of microbial killing, involving myeloperoxidase (MPO) generation of hypochlorous acid from hydrogen peroxide in the presence of Cl^-^. Studies have explored killing attributable to oxidative mechanisms in CF neutrophil phagosomes, revealing impaired chlorination of bacteria (17, 28–31). In turn, non-oxidative mechanisms of microbial killing involve cytoplasmic granules that release their content of antimicrobial peptides and enzymes directly into the phagosome (32). Protease and peroxidase activity is supported by changes in phagosomal pH, which is governed by protons and ions that compensate the electrogenic charge incurred by NOX2 activation (33).

The aim of this study was to shed further light on conditions prevailing in CF neutrophil phagosomes, with focus on intra-phagosomal degranulation and pH. Our data demonstrate prolonged alkalinization of phagosomes and impaired bactericidal processes, a defect rectified by ivacaftor therapy of PWCF with the *Gly551Asp* mutation.

## Materials and Methods

### Chemicals and Reagents

All chemicals and reagents were of the highest purity available and were purchased from Sigma Aldrich Ireland unless indicated otherwise.

### Study Design

PWCF were recruited from the Beaumont Hospital Cystic Fibrosis Clinic. Ethical approval was received from the Beaumont Hospital Ethics Board (REC reference # 14/98) and informed consent obtained from all study participants. Clinical demographics of all participants are presented in **Table 1**. To assess the effect of ivacaftor on neutrophil function, PWCF with the genotype *Gly551Asp/Phe508del* receiving 150 mg ivacaftor from Vertex Pharmaceuticals twice daily (*n =* 10, mean age 28.3 ± 8.17, FEV_1_ 53.1 ± 27.27% predicted) were recruited. Healthy control volunteers were age and sex-matched, had no respiratory symptoms and were not receiving medication.

**Table 1:** Clinical Demographics of patients and healthy controls recruited to this study.

Clinical demographic Parameter	Healthy controls	CF Genotype single *Phe508del* copy	CF Genotype *Phe508del/Phe508del*	CF ivacaftor therapy
No. of Subjects	28	18	10	10
Age, years (Mean ± SD)	30.06 ± 4.91	29 ± 7.1	28.2 ± 1.0	28.3 ± 8.17
Gender (Female/Male)	19/16	22/28	6/4	4/7
FEV1 (% predicted) (Mean ± SD)	104.33 ± 6.2	61.31 ± 23.07	59.2 ± 5.0%	53.1 ± 27.27
BMI (kg/m^2^)	23.8 ± 0.72	22.1 ± 2.75	22.04 ± 2.13	20.85 ± 1.72

Definitions of abbreviations: SD, Standard deviation; FEV1, forced expiratory volume in the first second; BMI, Body Mass Index.

### Neutrophil Isolation

Neutrophils were isolated as previously described (34). Cells were resuspended in phosphate buffered saline (PBS) containing 5 mM glucose (PBSG) unless specified otherwise. Purity of isolated neutrophils was validated by flow cytometric analysis using a monoclonal antibody against CD16b and was greater than 96% (35, 36). Neutrophil viability was assessed by Trypan Blue exclusion or by MTT (3-(4,5-dimethylthiazol-2-yl)-2,5-diphenyl tetrazolium bromide) assay and found to be >98%.

### Neutrophil Phagosome Isolation

The major steps in the experimental procedure are as previously outlined (37). In brief, a neutrophil suspension (1x10^8^ cells in PBSG) was rapidly stirred in a thermostatically controlled oxygen electrode chamber (Rank Brothers Ltd) with 2x10^10^ IgG coated latex particles (0.81 µM in diameter, Difco Laboratories). Phagocytosis was allowed to proceed for 8 min, and then stopped in ice cold PBSG. The cells were centrifuged (500 xg/10min/4°C) and to the pellet of neutrophils 1mM Diisopropyl phosphorofluoridate (DIFP) was added, whirly mixed and left on ice for 10 min. Cells were then suspended in 3 ml Break Buffer (10 mM KCl, 3 mM NaCl, 2 mM MgCl_2_, imM EDTA, 1 mM ATP, 20 mM Pipes, pH 7.2) containing protease inhibitors (10 mg/ml leupeptin, TLCK, pepstatin A and aprotinin) and 11.2% (w/w) sucrose. Cells were transferred to a cavitation chamber and brought to 400 psi with N_2_ gas for 20 min to achieve cell lysis and to obtain intact phagosomes. The homogenate was mixed with 60% (w/w) sucrose, and overlaid with 33% (w/w) then 11.2% (w/w) sucrose and centrifuged (20,000 g/30min/4°C) in a Sorvall SS3 centrifuge with swing out rotor. Phagosomes containing latex particles were harvested at the interface between the 11.2% (w/w) and 33% (w/w) sucrose, the concentration of sucrose was determined using a refractometer (B&S Abbe), and diluted to 11.2% (w/w). The suspension was then centrifuged (10,000g/10min/4°C) and the neutrophil phagosome pellet resuspended in PBSG. As an alternative approach, phagosomes were isolated following engulfment of 2.8 µm-sized IgG-coated Dynabeads magnetic beads (Thermo Fisher Scientific).

### Flow Cytometry Experiments

Flow cytometry was carried out to evaluate the membrane expression of CD16b as a measure of cell purity (38). Neutrophils were first fixed (4% (w/v) paraformaldehyde) and blocked (2% (w/v) BSA) for 30min at room temperature. After washing (PBS x 2) neutrophils (1x10^6^) were incubated with 1 µg/100 µl of mouse monoclonal anti-CD16b (Santa Cruz, Germany). Control samples were exposed to relevant non-specific isotype control IgG or secondary labelled antibody alone (FITC labelled bovine anti-mouse; Santa Cruz Biotechnology*).* For measuring degranulation into the phagosome, purified phagosomes were fixed with 4% (w/v) paraformaldehyde for 10 min, washed and blocked with 1% (w/v) BSA for 1 h, followed by incubation with 1µg/100µl mouse FITC-conjugated anti-CD66b or mouse phycoerythrin (PE)-conjugated anti-CD63 (BD, Biosciences). Controls included mouse PE IgG (control for mouse Mab anti-CD63) or mouse FITC IgM (control for mouse FITC anti-CD66b). For whole cell plasma membrane or phagosome membrane levels of HVCN1, samples were fixed, blocked and probed with a rabbit anti-HVCN1 antibody (Sigma, SAB3500536) for 1 h followed by incubation with an anti-rabbit FITC labelled IgG secondary antibody (Abcam, ab6717) and analyzed by flow cytometry. FITC Goat anti-Rabbit IgG served as a control. Samples were analyzed on a FACScalibur flow cytometer (Becton Dickinson, San Jose, CA, USA). At least 10,000 events were acquired and the mean fluorescence intensity (MFI) for each experiment was determined using BD CellQuest Pro software or FlowJo^®^ software.

### Phagosome pH Measurements

The pH of phagosomes of HC and CF neutrophils containing 2′,7′-Bis(2-carboxyethyl)-5 (6)- carboxyfluorescein acetoxymethyl ester (BCECF-AM, 5 μM, Life Technologies, Thermo Fisher) labelled *S. aureus* were assessed. Pasteurized *S. aureus* (1x10^9^ c.f.u) were pre-loaded with BCECF-AM dye (10 µM) for 30 min before removal of excess dye and opsonization with 1% (w/v) human IgG for 30 min. Neutrophils (2 x 10^7^) suspended in PBSG pH 7.4 were rapidly stirred in a 37°C thermostatically controlled oxygen electrode chamber. BCECF-AM labelled *S. aureus* (1 x 10^8^ c.f.u.) was added and aliquots removed at indicted time points up to 16 min. The intracellular pH measurements with BCECF were made by determining the pH-dependent ratio of emission intensity (detected at 535 nm) with the dye excited at 490 nm versus the emission intensity at 440 nm and correlated to pH values using an established pH standard curve (range pH 6–8).

As an alternative approach, fluorescence labelling of bacteria was also performed using the membrane-permeable pH indicator Carboxy SNARF-1 acetoxymethyl ester, acetate (Molecular Probes, Eugene, Oregon), which demonstrates a pKa of ~7.5, thus is useful for measuring pH changes between pH 7 and pH 8. Pasteurized *Candida albicans* (*C. albicans*) (1 x 10^8^) was preloaded with 50 µM Carboxy SNARF-1 in PBS for 30 min, before removal of excess dye. Purified neutrophils (2 X 10^7^) suspended in PBSG pH 7.4 were placed in the oxygen electrode chamber and 1 x10^8^ c.f.u. C. *albicans* added. Reaction aliquots were removed at indicated time points and analyzed in triplicate in a 96 well plate. The fluorescence emission was monitored at 580 and 640 nm using a Spectra Max M3 plate reader and correlated to pH values using an established pH standard curve in SNARF-1 Buffer (115 mM C_6_H_11_KO_7_, 15 mM NaCl, 5 mM MgCl_2_, 5 mM EGTA, 6 mM HEPES, 0.2 mM CaCl_2_, 1.3 mM Na_3_PO_4_, and 3 mM Na_2_HPO_4_) (range pH 6–8).

### SDS-Polyacrylamide Gel Electrophoresis and Western Blotting

Electrophoresis of samples was conducted according to Laemmli’s method (39). Denatured protein samples (20 ml) were resolved on 10 or 12.5% (w/v) resolving gel and 4% (w/v) stacking gel. SeeBlue Plus2 Prestained molecular mass markers (4 µl; Invitrogen) were loaded on each gel for determination of molecular weight. Gels were run in an ATTO AE6450 electrophoresis tank (ATTO Corporation, Tokyo, Japan) and electrophoresis was carried out for 60–90 min at 150V.

Following electrophoresis, proteins were transferred onto PVDF membrane at 150 mA for 60 min using a semidry blotting apparatus. Following transfer, membranes were blocked with 5% (w/v) non-fat powdered milk in PBS containing 0.1% (v/v) Tween-20 (PBST) for 1 h at room temperature. For immunological detection of the degranulated proteins in purified phagosomes, blots were incubated overnight at 4°C in blocking buffer containing either 1µg/ml rabbit anti-MPO (Novus Biologicals), rabbit anti-hCAP18 (Invitrogen) or goat anti-MMP9 (R&D Systems) antibody, respectively. Anti-human-IgG antibody served as a loading control. Relative secondary antibodies were all horseradish peroxidase (HRP) linked anti-goat or anti-rabbit (Cell Signalling Technology). Immunoreactivity was detected using Immobilon™ Western Chemiluminescent HRP- substrate (Millipore) solution using the G:BOX SynGene or ChemiDoc systems (Synoptics, UK; Bio-Rad, UK). Densitometry analysis was carried out using the GeneSnap or ImageLab programmes (Synoptics; Bio-Rad).

### Neutrophil Phagocytosis and Killing Assays

Phagocytosis assays were carried out as previously described with minor changes (40). In control experiments to evaluate equal phagocytosis of bacteria by HC and CF neutrophils, *S. aureus* (2 x 10^8^ bacteria) was resuspended in 1 ml of labelling buffer (50 mM Na_2_CO_3_, 100 mM NaCl, pH 9) containing 0.5 mg/ml of FITC and were incubated for 20 min at room temperature, pelleted by centrifugation (20,000x*g* for 10 min) and then washed x3 in 1 ml of PBS. FITC labelled bacteria were serum opsonized for 30 min and then washed with PBS. FITC labelled bacteria (1 x 10^8^ serum opsonized) and neutrophils were mixed at 37°C in a rapidly stirring oxygenated chamber at a 5:1 ratio. Aliquots were removed at 8 min and placed in 0.4% (v/v) Trypan Blue in PBS to quench extracellular and membrane adhered FITC labelled bacteria. Cells were then analyzed by flow cytometry for phagocytosed fluorescent bacteria as previously described (40). In a subset of experiments neutrophil phagocytosis in the presence of 100 µM ZnCl_2_ was assessed and found to be >97%.

Intraphagosomal killing was carried out as previously described (41). In brief, neutrophils (2 x 10^7^ cells) from PWCF or healthy controls were incubated at 37°C in PBSG in a stirring oxygenated chamber and serum-opsonized *S. aureus* (1 x 10^8^ c.f.u.) added. In a subset of reactions, neutrophils were suspended in PBSG in the presence or absence of ZnCl_2_ (100 µm) for 10 min at room temperature prior to the addition of serum-opsonized *S. aureus*. For direct enzyme mediated killing, bacteria were exposed to either NE (100 nM), MPO (10 μg/ml), α-defensin (2.5 μg/ml) or azurocidin (10 μg/ml) at 37°C. For all experiments, 100 μl aliquots were removed at indicated time points. Serial dilutions of the bacteria or bacteria/neutrophil suspensions were plated in triplicate on Luria-Bertani (LB) agar plates and incubated at 37°C. Viable bacterial c.f.u. were counted the following day. Control experiments included bacteria exposed to ZnCl_2_, with no effect observed. Bacterial viability was expressed as a percentage of bacterial counts at time 0 min, the latter representing 100% viability.

### Statistical Analysis

Results are expressed as mean ± standard error of the mean (SEM) of *n* separate biological replicates as stated in the figure legends. Statistical analysis was performed with GraphPad Prism (version 4.03 for Windows). For statistical comparison of small datasets (*n* < 6) Student’s *t* test was performed to determine *P* values (42). For larger datasets the D’Agostino and Pearson omnibus normality test was carried out to determine whether data was normally distributed. When normally distributed, groups were compared by Student’s *t* test, otherwise by the nonparametric Mann-Whitney *U* test. For comparison of three or more groups one-way ANOVA was performed. *P* values were considered statistically significant with *P <*0.05. Differential expression of proteins identified by proteomic analysis was defined as greater than 1.5-fold change in expression with *P <*0.05 or 1.2-fold with *P <*00.1.

## Results

### Impaired Phagosomal Killing by CF Neutrophils

**Figure 1:**
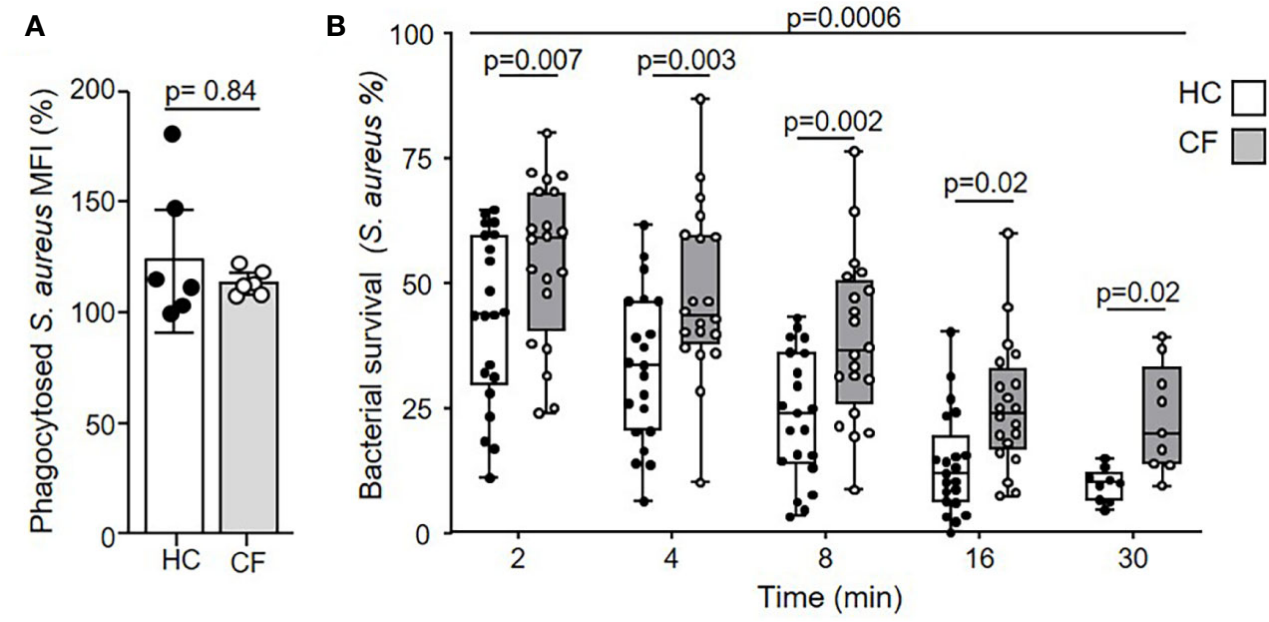
Impaired microbicidal activity of neutrophils from PWCF. **(A)** Phagocytosis of FITC labelled *S. aureus* was assessed by flow cytometry and expressed as mean fluorescence intensity (MFI). Healthy control (HC) and neutrophils of PWCF (*Phe508del* single copy) demonstrated equal levels of phagocytosis (n = 6 donors per group, paired t test). **(B)** Killing of *S. aureus* (0.5 x 10^7^ CFU/ml) by HC neutrophils was compared to CF cells donated by PWCF homozygous for the *Phe508del* mutation Killing rates between HC and CF neutrophils were significantly different (p = 0.0006) and reduced at each time point (n = 7 subjects per group, linear mixed effects model with post-hoc Holm-Šídák test).

Flow cytometry with fluorescent labelled serum opsonized bacteria confirmed equal phagocytosis of *S. aureus* by HC and CF neutrophils (**Figure 1A**). Intraphagosomal killing of *S. aureus* by neutrophils is a rapid process (34), and in the current study HC neutrophils successfully killed 68% of bacteria within 4 min (**Figure 1B**). The pattern of killing by neutrophils of PWCF homozygous for the common *Phe508del* mutation differed. The percentage of viable *S. aureus* post 8 min phagocytosis by CF neutrophils was 42%. At the same time point, HC neutrophils reduced bacterial viability to 23% (p = 0.002). As the kinetics of killing by CF and HC cells differed up to 30 min (p = 0.0006), a period of time coinciding with degranulation of cytoplasmic granules and changes in phagosomal pH (43, 44), alterations in these key processes in CF phagosomes was assessed.

### Dysregulated Degranulation of Antimicrobial Enzymes in CF Neutrophil Phagosomes Is Corrected by CFTR Potentiator Therapy

**Figure 2:**
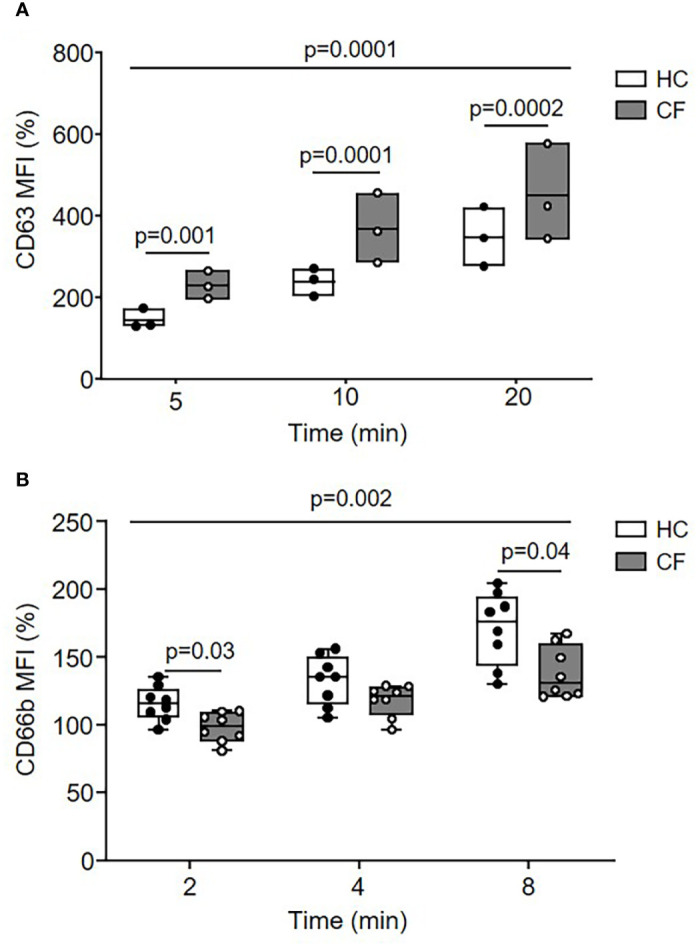
Dysregulated movement of granular proteins to phagosomes of neutrophils from PWCF. **(A, B)** Phagosomes were isolated following phagocytosis of IgG opsonized latex particles. Phagosomal membrane levels of CD63 **(A)** or CD66b **(B)** were quantified by flow cytometry and expressed as mean fluorescence intensity (MFI). Compared to HC, CF phagosomes (*Phe508del* single copy) displayed significantly increased levels of CD63 (n = 3 subjects per group, two-way ANOVA with post-hoc Šídák test) and significantly reduced levels of CD66b (n = 8 subjects per group, two-way ANOVA with post-hoc Šídák test). Data represent mean percentage change in fluorescence from time zero.

Disproportionate extracellular release of cytoplasmic granules into surrounding media in which CF neutrophils are bathed occurs in response to soluble stimuli (20, 45), however, the kinetics of intraphagosomal degranulation was undetermined. By flow cytometry of purified phagosomes following engulfment of IgG opsonized Dynabeads, degranulation of primary or secondary/tertiary granules was assessed measuring levels of phagosomal membrane CD63 or CD66b (46), respectively (**Figure 2**). Upregulation of CD63 to the phagosomal membrane was greatly increased in neutrophils of PWCF homozygous for the *Phe508del* mutation compared to HC cells (p = 0.001, p = 0.0001, and p = 0.0002, after 5, 10, or 20 min, respectively) (**Figure 2A**). In contrast, the level of CD66b was significantly decreased on CF phagosomal membranes compared to HC samples across the time course (p = 0.002) and at 8 min (p = 0.04) (**Figure 2B**).

**Figure 3:**
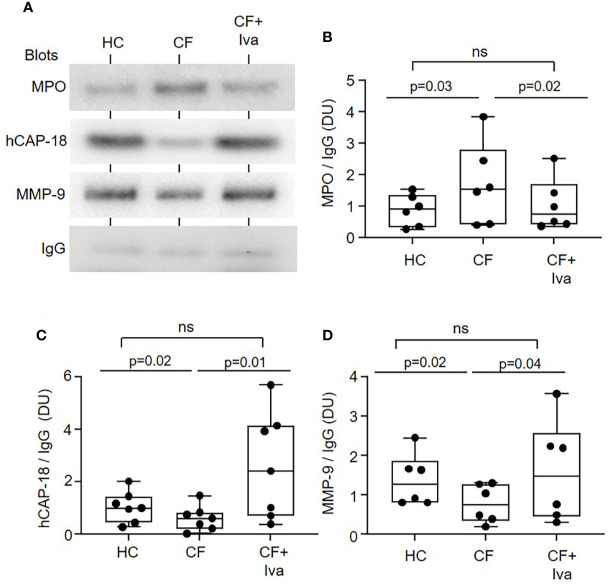
Altered levels of granular proteins in phagosomes of neutrophils from PWCF. **(A)** Purified phagosomes were lysed and levels of MPO, hCAP-18, or MMP-9, markers of primary, secondary, and tertiary granule degranulation respectively, were assessed by Western blotting. Compared to phagosomes of HC or PWCF with the *Gly551Asp* genotype receiving ivacaftor therapy (CF+Iva), CF phagosomes (*Phe508del* single copy) contained significantly increased levels of MPO **(B)**, and decreased levels of hCAP-18 **(C)** and MMP-9 **(D)**. Levels of MPO, hCAP-18 and MMP-9 were not significantly different between HC and CF+Iva (n = 6 or 7 subjects per group, mixed effects model with Tukeys’ *post hoc* test). The opsonin IgG was found equally expressed between the different phagosome types and was therefore used as evidence of equal phagocytosis and as a loading control. Band intensity for MPO, hCAP-18, and MMP-9 was quantified by densitometry [expressed as densitometry units (DU)] and normalized to IgG.

To confirm disturbed degranulation, an alternative approach was taken whereby neutrophil phagosmes containing IgG coated latex particles were purified by sucrose density ultracentrifugation and the phagosomal content of granule proteins quantified by immunoblotting (**Figure 3A**). Equivalent immunoband intensity in response to anti human-IgG antibody in HC and CF samples, confirmed equal levels of phagocytosis. Levels of intraphagosomal MPO from primary granules was significantly increased (p = 0.03) after 8 min phagocytosis (**Figures 3A, B**), but in contrast, levels of hCAP-18 from secondary (p = 0.02) (**Figures 3A, C**) and MMP-9 from tertiary granules (p = 0.02) (**Figures 3A, D**) were significantly decreased in CF phagosomes compared to HC samples. Further experiments evaluated the effect of CFTR potentiator therapy on intraphagosomal degranulation. Results revealed that the level of MPO in neutrophil phagosomes of PWCF with the *Gly551Asp* mutation, who were receiving ivacaftor, were increased almost on par to HC cells (**Figures 3A, B**). Moreover, statistical analysis revealed that CF phagosomes of neutrophils donated by PWCF receiving CFTR potentiator ivacaftor therapy illustrated phagosomal levels of hCAP-18 and MMP-9 similar to control cells, and increased compared to homozygous Δ*F508* patients post 8 min phagocytosis (p = 0.01 and p = 0.04, respectively) (**Figures 3C, D**).

Collectively, these results indicate changes in phagosomal granule content of neutrophils from individuals with CF, which could contribute significantly to impaired antimicrobial activity. However, phagosomal pH also plays a significant role in protease activity and microbial killing, and was therefore explored next.

### Increased Alkalinity of Neutrophil Phagosomes in CF

**Figure 4:**
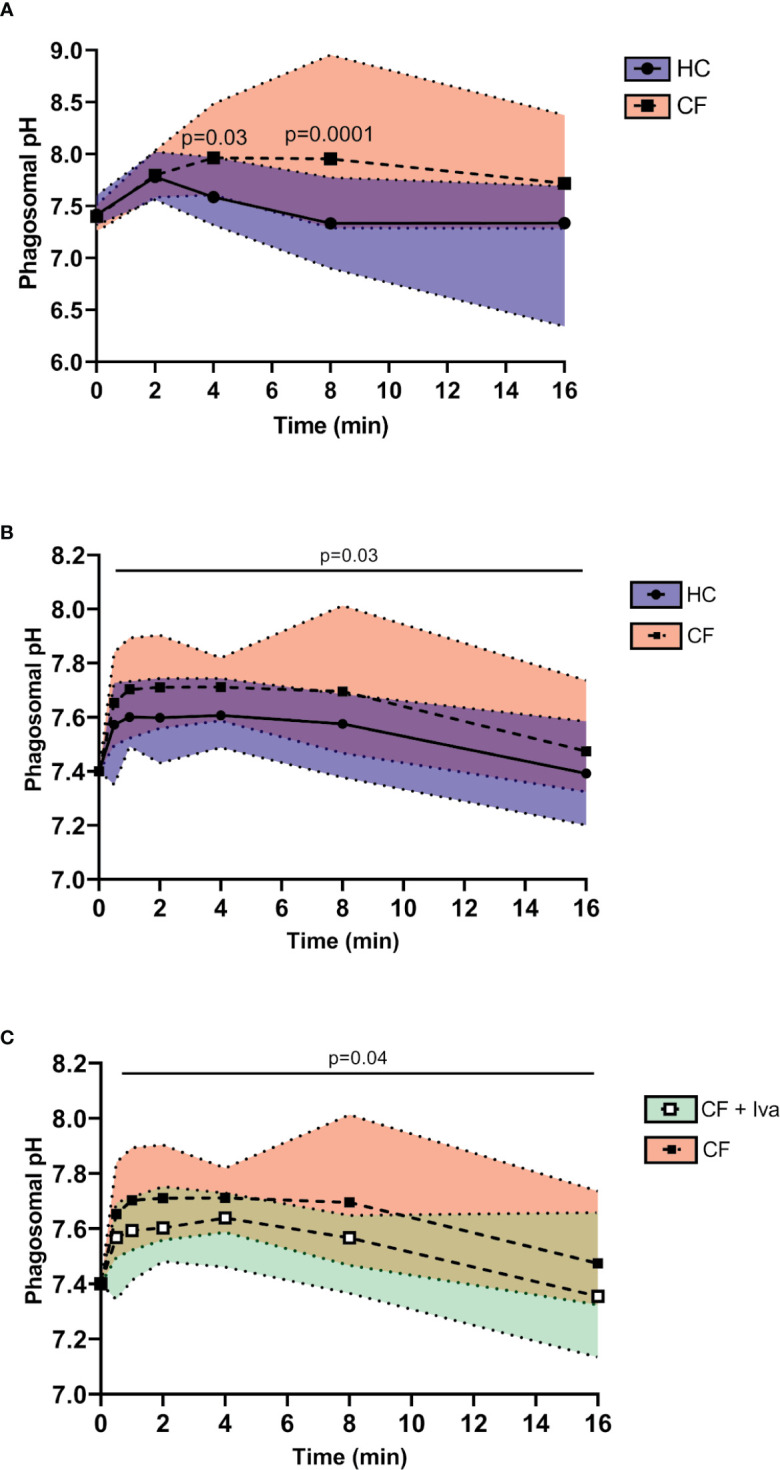
Prolonged alkalinity of CF phagocytic vacuoles. **(A)** Phagosomal pH was assessed using the pH sensitive fluorescent probe BCECF-AM. Significantly increased phagosomal pH was measured in CF neutrophils compared to HC post phagocytosis of probe labelled *S. aureus* (n = 3 subjects per group, two-way ANOVA with Šídák *post hoc* test). **(B)** Time course of phagosomal pH changes assessed using SNARF labelled *C. albicans*, phagocytosed by neutrophils of HC, or *Phe508del* homozygous CF neutrophils. Phagosomes of *Phe508del* CF neutrophils demonstrated significantly increased pH (n = 3 subjects per group, mixed effects model with Tukeys’ *post hoc* test). **(C)** Time course of phagosomal pH changes assessed using SNARF labelled *C. albicans*, phagocytosed by neutrophils of *Phe508del* homozygous CF neutrophils (as per data presented in panel **B**) or neutrophils from *Gly551Asp* PWCF receiving ivacaftor therapy (CF+Iva). Phagosomes of *Phe508del* CF neutrophils demonstrated significantly increased pH (n = 3 subjects per group, mixed effects model with Tukeys’ *post hoc* test).

Upon phagocytosis of microbial pathogens, NOX2 activity results in membrane depolarization. Compensatory ion movement into the phagosome, including proton (47) and K^+^ influx (34) impact upon the pH (44). Reduced cytosolic pH has previously been recorded in resting (48) and activated CF neutrophils (19) but phagosomal pH was not explored. Phagosomes of HC neutrophils containing BCECF-AM-labelled *S. aureus* demonstrated significant alkalinisation with a mean maximum pH 7.78 recorded after 2 min phagocytosis (range 7.71–8.02) (**Figure 4A**). This rise in pH was rapid and was followed by a fall in pH to 7.3 (range 6.9–7.8) at 8 min.

This increase in pH is as previously described, albeit at a lower level (44, 47). By contrast, the pH in phagosomes of neutrophils of PWCF homozygous for the *Phe508del* mutation at 8 min post phagocytosis was significantly elevated to a mean value of 8.0 (range 7.3–8.95) (p = 0.0001).

As fluorescein saturates at approximately pH 8, and may become bleached within the phagosme (43), SNARF labelled *C. albicans* was alternatively used to determine the phagosomal pH (47). Changes in pH were tracked immediately upon engulfment and up to 8 and 16 min post phagocytosis (**Figure 4B**). In HC neutrophils the mean maximum pH of 7.6 (range 7.43–7.74) was obtained at 2 min post phagocytosis and was maintained up to 4 min. Moreover, an elevation in pH in phagosomes of neutrophils of PWCF homozygous for the *Phe508del* mutation was observed where the mean maximum pH post-phagocytosis was 7.71 (range 7.56–7.9). This elevated pH in CF phagosomes was maintained over 8 min. By two-way ANOVA with Šídák *post hoc* test a significant increase above that of the HC phagocytic pH values was recorded across the time course (p = 0.03). Of note, at the 16 min phagocytosis time point, no difference in pH was observed. Moreover, the alkalinization observed in phagosomes of *Phe508del* CF neutrophils was in contrast to that recorded in neutrophil phagosomes of PWCF with the *Gly551Asp* mutation who were receiving ivacaftor. Statistical analysis revealed that CF neutrophil phagosomes donated by PWCF on ivacaftor therapy illustrated mean maximum pH levels of 7.64 at 4 min (range 7.46–7.73), which was significantly decreased compared to homozygous *Phe508del* patients samples (p = 0.04) (**Figure 4C**), and similar to HC values (**Figure 4B**).

Collectively, these results indicate changes in the phagosome of neutrophils from individuals with CF, with the impact of altered pH on bactericidal processes next explored.

### Prolonged Alkinalization of CF Phagosomes Impacts on pH Dependent Anti-Microbial Killing

**Figure 5:**
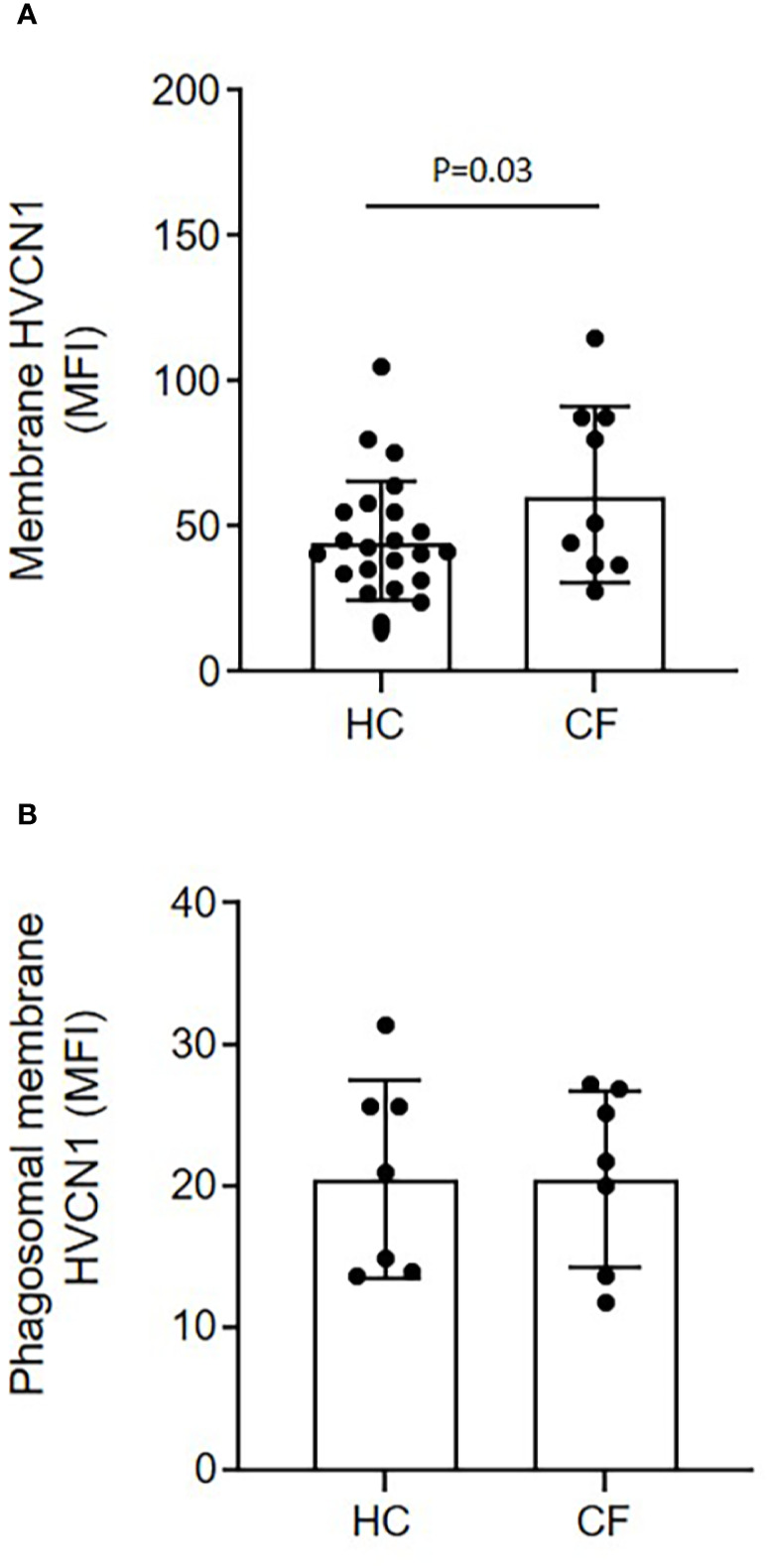
Equal expression of the H^+^ proton channel HVCN1 on phagocytic membranes of neutrophils of HC and PWCF. Neutrophil membrane levels of HVCN1 were quantified by flow cytometry and expressed as mean fluorescence intensity (MFI). **(A)** CF neutrophils demonstrated increased plasma membrane HCVN1 expression compared to HC samples (n = 23 and n = 9, respectively, paired t test). **(B)** Phagosomes were isolated following 8 min phagocytosis of IgG opsonized latex particles. CF phagosomes demonstrated equal levels of HVCN1 compared to HC (n = 7 per group, paired t test, p = 0.65).

**Figure 6:**
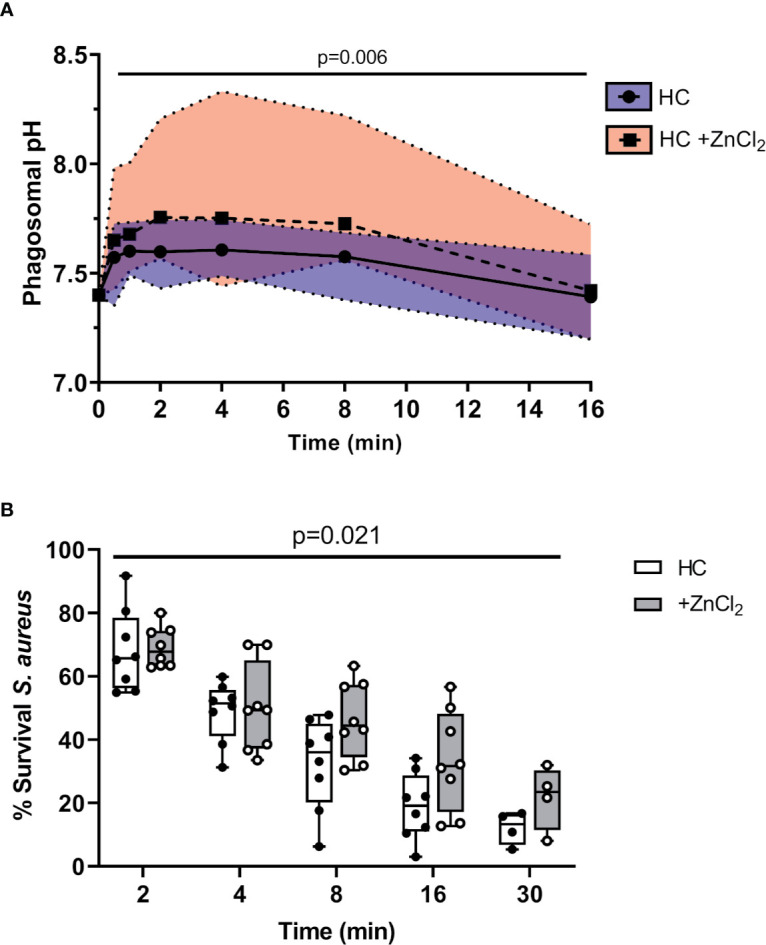
Altered pH negatively impacts killing of phagocytosed bacteria. **(A)** Time course of phagosomal pH changes assessed using SNARF labelled *C. albicans* phagocytosed by neutrophils of HC ± ZnCl_2_ (100 µM). Phagosomes of ZnCl_2_ treated HC neutrophils demonstrated significantly increased pH (n = 3 subjects per group, mixed effects model with Tukeys’ *post hoc* test). **(B)** Time course of *S. aureus* killing (0.5 x 10^7^ c.f.u./ml) by HC neutrophils treated with 100 µM ZnCl_2_ is significantly reduced compared to untreated cells (n = 8 biological repeats, two-way ANOVA with post-hoc Šídák test).

Ensuing experiments investigated the impact of altered phagosomal pH. In *Hvcn1^-/-^* mice, or ZnCl_2_ treated cells, the vacuolar pH becomes extremely alkaline (47). Although CF neutrophil plasma membranes expressed significantly higher levels of HVCN1 compared to HC samples (p = 0.03), possibly due to the primed state of circulating CF cells, phagosomal membrane levels were found to be similar between the two cell types (**Figures 5A, B**, respectively). Thus, inhibition of this channel by inclusion of ZnCl_2_ was performed so as to understand the impact of elevated pH on phagosomal microbial killing. By use of SNARF labeled *C. albicans*, and inhibition of HVCN1 by 100 µM ZnCl_2_, the phagosome was alkalinized, with a mean pH of 7.75 (range 7.57–8.2) observed at 2 min (**Figure 6A**). The elevation observed in pH following inhibition of HVCN1, was in line with phagosomes of neutrophils of PWCF homozygous for the *Phe508del* mutation, where the mean maximum pH post-phagocytosis was 7.71 (range 7.56–7.9) (**Figure 4B**).

Subsequent killing assays demonstrated that inhibition of HVCN1 by inclusion of ZnCl_2_ significantly decreased intraphagosomal killing of *S. aureus* by HC neutrophils (**Figure 6B**). The kinetics of killing by ZnCl_2_ treated and untreated cells differed across the entire time course up to 30 min (p = 0.02). The percentage of viable *S. aureus* post 8 min phagocytosis by untreated neutrophils was 30%, and in contrast at the same time point, ZnCl_2_ treated cells reduced bacterial viability to 45%, a killing ability similar to CF neutrophils recorded in **Figure 1B**.

**Figure 7:**
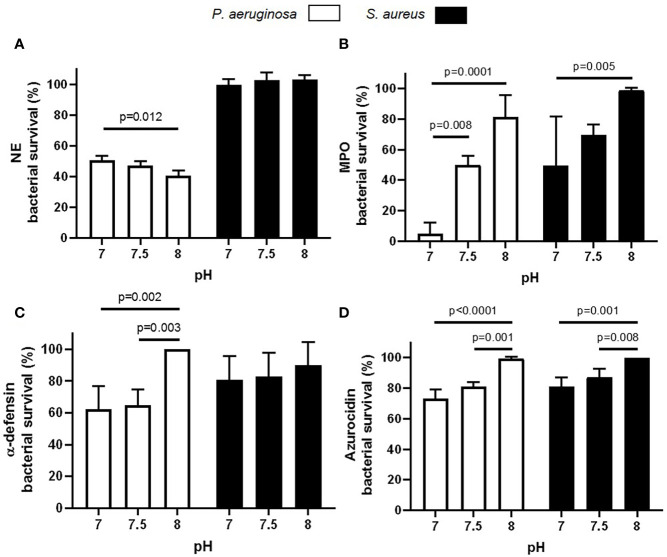
Altered pH impacts enzymatic killing of bacteria. **(A–D)** Variations in pH significantly decrease NE (**A**; 100 nM), MPO (**B**; 10 μg/ml), α-defensin (**C**; 2.5 μg/ml), and azurocidin (**D**; 10 μg/ml) killing of *P. aeruginosa* or *S. aureus* (n = 3 biological repeats, two-way ANOVA, post-hoc Šídák test).

As the highest mean maximum pH recorded in HC neutrophil phagosomes at 8 min post phagocytosis using BCECF-AM was 7.3, and in CF phagosomes at the same time point was pH 8.0, we compared the killing ability of neutrophil antimicrobial components over this pH range (**Figure 7**). This set of experiments investigated the impact of altered pH on bacterial killing employing the archetypal CF infecting microbes *P. aeruginosa* and *S. aureus.* Results revealed a significant decrease in *P. aeruginosa* viability, but not *S. aureus* viability, when incubated with NE. After 8 min NE incubation, maximal killing of *P. aeruginosa* occurred at pH 8.0 (p = 0.01) (**Figure 7A**). Results also revealed a significant decrease in *P. aeruginosa* and *S. aureus* viability when incubated with MPO at pH 7.0 compared to pH 8.0 (p = 0.0001 and p = 0.005, respectively), with ~5 and 50% bacterial survival recorded after 8 min at pH 7.0, respectively (**Figure 7B**). Although α-defensins demonstrated little effect against *S. aureus* at any pH, purified HNP1-4 successfully reduced *P. aeruginosa* survival by 36% at pH 7.5, a level significantly increased compared to pH 8.0 (p = 0.003) (**Figure 7C**). Moreover, a significant decrease in *P. aeruginosa* and *S. aureus* viability was recorded after 8 min incubation with azurocidine at pH 7.5 compared to pH 8.0 (p = 0.001 and p = 0.008, respectively) (**Figure 7D**). Overall, these results indicate that increased phagosomal pH is supportive of serine protease NE bacterial killing, but leads to reduced bacterial killing by major components of neutrophil primary granules including α-defensins and azurocidine, but most noticeably MPO.

## Discussion

Studies indicating functional and signaling changes in CF neutrophils, that could impact clinical prognosis and lung disease severity, has gained much interest. In the present study, we report significant prolonged alkalinization of CF neutrophil phagosomes, engendering inadequate microbial killing conditions by MPO, α-defensins and azurocidine. In PWCF, ivacaftor treatment corrects the recorded dysregulated levels of degranulation and functions to normalize phagosomal pH to that of healthy controls.

A lack of CFTR function or presence in myeloid cells can lead to a pro-inflammatory response of both circulating and airway neutrophils, with significant overproduction of neutrophil chemoattractants (49). Confusion as to why recruited neutrophils fail to kill invading microbes continues, with the bacterium *Burkholderia cenocepacia* and *Mycobacterium abscessus* causing severe lung infections in patients with CF. Signaling mechanisms between opsonins and neutrophil receptors required for bacterial phagocytosis can be affected within the CF airways, as high protease activity (50, 51) can cleave CXCR1 (52), Fcγ receptors and iC3b (53, 54). However, similar rates of phagocytosis by purified HC and CF blood neutrophils has been reported (31) and also observed in the current study. Although small, a significant reduction was observed in the intraphagosomal killing ability of CF neutrophils against *S. aureus*. Previous studies have also demonstrated impaired CF neutrophil killing of *P. aeruginosa* (17), *Burkholderia cenocepacia* and *Haemophilus influenza*e (55), which may allow the bacteria to establish an initial foothold in the lung of PWCF.

Studies investigating mechanisms that may lead to altered activity of CF neutrophils have demonstrated increased intracellular concentrations of calcium (55, 56), which correlate with significantly reduced oxidase activity and impaired formation of antimicrobial extracellular traps (55). Impaired microbial killing is also linked to diminished MPO mediated phagosomal HOCl production and chlorination of phagocytosed bacteria (17, 29). Two Cl^-^ ion channels (ClCs), ClC-3 and CFTR, are associated with transport of Cl^-^ within the neutrophil and the phagosome (17, 57). Moreover, the influx of protons to the phagosomal lumen by V-ATPase has been demonstrated to facilitate transport of Cl^-^ ions by ClCs including CFTR (58). The apparent intrinsic defect in PWCF was further supported by data demonstrating that bacterial glutathione sulfonamide formation, a HOCl product, is reduced in CF neutrophils (31). In the current study we observed increased primary granule degranulation and MPO accumulation in the CF phagosome, yet decreased bacterial killing, supporting the concept of unsuitable conditions in the CF phagosome for optimal MPO peroxidase activity. The exact mechanism leading to increased primary granule release by CF cells most likely involves increased Rac2 activation (59). Moreover, impaired GTP-Rab27a activation in CF blood neutrophils has been shown to decrease secondary and tertiary granule degranulation to the outside of the cell in response to soluble stimuli (20). Of interest, by proteomic analysis of CF neutrophil plasma membranes, dysregulated degranulation has been observed in neutrophils donated by patients during a CF exacerbation and in the same individuals when stable (20), thus suggestive of an intrinsic impairment. In the present study, differences in the degranulation pattern of these two granule types into the CF phagosome may also contribute to impaired bacterial killing, particularly as hCAP-18 possesses antimicrobial activity against both *S. aureus* and *P. aeruginosa* (60).

Confirmation of the importance of MPO as part of the neutrophil’s armory to fight infection was confirmed by use of MPO knockout mice, in which killing of *Candida albicans* was defective (61). Evidence with *S. aureus* points to the anti-microbial process being strongly dependent on MPO (62). Acidification of phagosomes has been proposed to play a key role in the microbicidal function of phagocytes. Indeed MPO peroxidase activity is most optimal at acid pH, and as far back as the 1970’s it was shown that the process of ^36^Cl^-^ incorporation to an insoluble fraction decreased as the pH was elevated from 4 to 7.4 (63, 64). Subsequently however, in 1982 Segal and co-workers employed pH indicator fluorescein conjugated to *S. aureus* and following phagocytosis, measured early pH changes within the phagosome (44). Results indicated a transient increase in pH to 7.8–8.0 within the first 2 min, which was followed by a slow fall to 6.0–6.5 after 2 h. A further study using similar fluorometric techniques later confirmed these observations (43). The pattern of pH within phagosomes was clearly different in neutrophils of patients with chronic granulomatous disease, that lack NOX2 activity, or with control neutrophils in anaerobic conditions where the pH fell rapidly from 7.4 to 6.6 within the first two minutes (44). Accordingly, abnormal acidification can be averted *in vitro* by the use of lysosomotropic weak bases or the vacuolar‐type H^+^ pump inhibitor concanamycin A (65).

During the described initial rise in pH, MPO can act as a catalase rather than a peroxidase (66), a role that may dominate under the prolonged alkaline conditions observed in the CF phagosome thus leading to impaired bacterial killing. Eight minutes post phagocytosis we recorded a difference in the phagosomal pH between HC and CF neutrophils, a time corresponding to 80% bacterial killing by HC cells, and twice the number of bacteria surviving in CF neutrophils. The prolonged rise in pH at ~8.0 would provide an optimal milieu for the granule proteases NE and cathepsin G, which are active at this pH (67) but would be less supportive of azurocidine and α-defensin, as demonstrated here and by others (68, 69).

In alveolar macrophages it has been proposed that CFTR contributes to alterations in lysosomal pH, as lysosomes from CFTR-null macrophages failed to acidify (70). However, further studies have indicated that phagolysosomal acidification in macrophages may not be dependent on CFTR channel activity (71, 72). The difference in phagosomal pH in *Hvcn1^-/-^* neutrophils, or those in which the proton channel has been inhibited by inclusion of ZnCl_2_ in the current study, provides evidence that HVCN1 compensates the electrogenic charge incurred upon NOX2 activation (47). Moreover, it has been shown that killing of *S. aureus* by *Hvcn1^-/-^* bone marrow cells is impaired (33) and related to this, in the current study *S. aureus* killing ability of CF neutrophils or control neutrophils treated with ZnCl_2_, were significantly decreased. Collectively, these results were suggestive of altered HVCN1 expression on membranes of CF neutrophils. However, excessive alkalinization of phagosomes of CF cells is most likely not due to alterations in the expression of HVCN1, as equal levels of the proton channel were detected on healthy control and CF neutrophil membranes. Delayed proton channel activity may be one cause for the observed altered pH, however, a recent study supports an interesting and possible alternative explanation for the alter phagosomal pH observed in CF (73). Critical illness is often characterized by immune dysregulation and systemic complement activation, and C5a exposure prior to neutrophil-bacterial interactions was shown to lead to impairment of phagosomal acidification (73). In CF respiratory fluids, levels of C5a correlate negatively with FEV1% predicted (74), but the influence of C5a on neutrophil phagosomal pH within the CF airways may not be relevant as serine protease cleavage of C5aR can inactivate C5a-induced signaling ability (75). Perhaps more relevant is the possible interaction between blood neutrophils and systemic C5a levels, prior to neutrophil migration to the CF airways and bacterial interaction. This is a noteworthy concept that requires further investigation.

CFTR potentiators and correctors can restore much of the function of the majority of CFTR variants. Therapy leads to improved airflow and normalized airway surface liquid composition, resulting in reduced inflammation and minimized airway remodelling. Interestingly, our study found a significant difference in phagosomal conditions of neutrophils of PWCF with the *Gly551As*p mutation receiving ivacaftor therapy compared to PWCF with different mutations. In this regard, we observed normalized pH and corrected degranulation pattern of neutrophils from patients who were receiving ivacaftor for the preceding two years compared to corrector treatment naïve individuals. As ivacaftor was shown to improve CFTR function, but also reduce levels of circulating inflammation (56), the current study has not established whether the observed differences in CF phagosomes is due to CFTR dysfunction or the underlying inflammatory burden, and this is a limitation of the study. Nevertheless, as approval for CF modulator therapies continues to be granted to younger cohorts who will receive these treatments before the onset of structural and inflammatory changes in their airways (76), dysregulated neutrophil function may be less a problem in the future. In such cohorts, it is possible that the impaired neutrophil processes reported in this study including altered pH may not emerge, as airway inflammatory burden will not manifest to the same extent.

## Data Availability Statement

The raw data supporting the conclusions of this article will be made available by the authors, without undue reservation.

## Ethics Statement

The studies involving human participants were reviewed and approved by Beaumont Hospital Ethics Board Beaumont Hospital, Dublin 9, Ireland. The patients/participants provided their written informed consent to participate in this study.

## Author Contributions

ER, NM, EH, KP, and NB conceived and planned the study design, designed experiments, performed quality assurance, interpreted the data, and wrote the manuscript. NB, PH, KM, KP, CF, and LS carried out experiments. MM, KP, and KM performed statistical analysis. NM, PH, CG, OM, and FG contributed to patient accrual and clinical data collection. ER and NM share joint senior authorship. All authors contributed to the article and approved the submitted version.

## Funding

We would like to acknowledge our funding sources, including the Health Research Board Ireland (MRCG-2018-04) (ER), the US Cystic Fibrosis Foundation (NM), Charitable Infirmary Charitable Trust (NM), Beaumont Hospital Foundation, Dublin, Ireland, and the Programme for Research in Third Level Institutes (PRTLI) administered by the Higher Education Authority (NM).

## Conflict of Interest

The authors declare that the research was conducted in the absence of any commercial or financial relationships that could be construed as a potential conflict of interest.
